# Supplementary material for: Intensive Rehabilitation Program in Older Adults with Stroke: Therapy Content and Feasibility—Preliminary Results from the BRAIN-CONNECTS Study
Source: Int J Environ Res Public Health. 2023 Mar 7;20(6):4696. doi: 10.3390/ijerph20064696 (PMC10048316; doi:10.3390/ijerph20064696)
Supplement: Supplementary file 1 [file ijerph-20-04696-s001.zip › ijerph-2205752-supplementary/Supplementary Material. Table S1. Data Collection Notebook.pdf]

Supplementary Material. Table S1: Data collection Notebook

PHYSICAL THERAPY Data Collection

Patient's name: ____ ID: _______________ Rehabilitation Start Date: ___ /__ /____ Patient's nº: _____

PHYSICAL THERAPY		Session nº ___	Session nº ___	Session nº ___	Session nº ___	Session nº ___
	Date					
	Morning / Afternoon					
	Therapist's name					
Activities	Passive mobilizations of affected extremities.	*Time*	*Time*	*Time*	*Time*	*Time*
	Auto-Assisted exercises of affected extremities.	*Time*	*Time*	*Time*	*Time*	*Time*
	Active exercises of affected extremities.	*Time*	*Time*	*Time*	*Time*	*Time*
	Balance training and stepping.	*Time*	*Time*	*Time*	*Time*	*Time*
	Trunk control training.	*Time*	*Time*	*Time*	*Time*	*Time*
	Body-weight supported treadmill training.	*Time*	*Time*	*Time*	*Time*	*Time*
	Standing training – Standing frame.	*Time*	*Time*	*Time*	*Time*	*Time*
	Stairs, ramps and obstacles.	*Time*	*Time*	*Time*	*Time*	*Time*
	Gait training.	*Time*	*Time*	*Time*	*Time*	*Time*
	Transfer training.	*Time*	*Time*	*Time*	*Time*	*Time*
	Aerobic training.	*Time*	*Time*	*Time*	*Time*	*Time*
	Coordination and integration exercises of the affected side.	*Time*	*Time*	*Time*	*Time*	*Time*
	**Total time/day**					
	Not attendance? – Reason					
	Early termination? – Reason					
	Comment/adverse events during session					

OCUPATIONAL THERAPY Data Collection

Patient's name: ______________________________ ID: ____________ Rehabilitation Start Date: ___ /__ /____ Patient's nº: _____

OCUPATIONAL THERAPY			Session nº ___	Session nº ___	Session nº ___	Session nº ___	Session nº ___
		Date					
		Morning / Afternoon					
		Therapist's name					
Activities	Upper Limb movement patterns, coordination, and dexterity training.		*Time*	*Time*	*Time*	*Time*	*Time*
	Task-specific training.		*Time*	*Time*	*Time*	*Time*	*Time*
	Active-assisted tasks upper limb weight support.		*Time*	*Time*	*Time*	*Time*	*Time*
	Postural control and orthosis use for affected upper limb.		*Time*	*Time*	*Time*	*Time*	*Time*
	Constraint Induced Movement Therapy (CIMT).		*Time*	*Time*	*Time*	*Time*	*Time*
	Mirror therapy.		*Time*	*Time*	*Time*	*Time*	*Time*
	Virtual reality for upper limb movement training.		*Time*	*Time*	*Time*	*Time*	*Time*
	Somatosensory rehabilitation.		*Time*	*Time*	*Time*	*Time*	*Time*
	Cognitive and perceptual tasks.		*Time*	*Time*	*Time*	*Time*	*Time*
	Activities of daily live training.		*Time*	*Time*	*Time*	*Time*	*Time*
	Stroke education.		*Time*	*Time*	*Time*	*Time*	*Time*
		**Total time/day**					
		Not attendance? – Reason					
		Early termination? – Reason					
		Comment/adverse events during session					

SPEECH THERAPY Data Collection

Patient's name: ______________________ ID: __________ Rehabilitation Start Date: ___/__/___ Patient's nº: _____

SPEECH THERAPY			Session nº ___	Session nº ___	Session nº ___	Session nº ___	Session nº ___
		Date					
		Morning / Afternoon					
		Therapist's name					
Activities	Verbal and written comprehension.		*Time*	*Time*	*Time*	*Time*	*Time*
	Oral production (articulation, volume, tone and prosody).		*Time*	*Time*	*Time*	*Time*	*Time*
	Phonological processing.		*Time*	*Time*	*Time*	*Time*	*Time*
	Morphosyntax's exercises.		*Time*	*Time*	*Time*	*Time*	*Time*
	Functional communication.		*Time*	*Time*	*Time*	*Time*	*Time*
	Lexical evocation (free, phonetic phonological and semantic).		*Time*	*Time*	*Time*	*Time*	*Time*
	Lingual praxis.		*Time*	*Time*	*Time*	*Time*	*Time*
	Cognition (memory, reasoning, logical reasoning and abstraction).		*Time*	*Time*	*Time*	*Time*	*Time*
	Respiratory Muscle Training (Orygen Dual Valve©).		*Time*	*Time*	*Time*	*Time*	*Time*
	Sensitive and gustatory stimulation.		*Time*	*Time*	*Time*	*Time*	*Time*
	Neuromuscular Electrical Stimulation (Vital Stym©).		*Time*	*Time*	*Time*	*Time*	*Time*
		**Total time/day**					
		Not attendance? – Reason					
		Early termination? – Reason					
		Comment/adverse events during session					
