# Supplementary material for: Intensive Rehabilitation Program in Older Adults with Stroke: Therapy Content and Feasibility—Preliminary Results from the BRAIN-CONNECTS Study
Source: Int J Environ Res Public Health. 2023 Mar 7;20(6):4696. doi: 10.3390/ijerph20064696 (PMC10048316; doi:10.3390/ijerph20064696)
Supplement: Supplementary file 1 [file ijerph-20-04696-s001.zip › ijerph-2205752-supplementary/Supplementary Material. TableS2. Description of activities.pdf]

Supplementary Material. Table S2: Description of activities

PHYSICAL THERAPY ACTIVITIES

ACTIVITIES	DESCRIPTION OR EXAMPLES
Passive mobilizations of affected extremities	It consists of passive mobilizations by the physiotherapist in all possible directions of joint movement the lower and/or upper limb without contraction of the muscle by the patient. The objective is to maintain the range of joint movement, prevent adhesions and retractions.
Auto-Assisted exercises of affected extremities	Exercises or mobilizations of the affected upper and/or lower limb assisted by the patient himself with the active collaboration of his healthy extremities. It can be done using simple pulleys, pedals, ropes, etc. This exercise aims to facilitate joint movements by integrating them into the body schema and maintain the full range of joint movement.
Active exercises of affected extremities	Active exercises with muscle contraction of the upper or lower limb performed by the patient himself in different positions (supine, sitting, standing or quadruped). This exercise promotes to recover or maintain muscle function, strength and muscle tone and prevent muscle atrophy.
Balance training and stepping	Sitting or standing exercises with the aim of training balance. It can be trained in different body positions or different platforms. For example: static or dynamic balance with unstable planes, monopodal standing, tandem, going up and down steps, etc.
Trunk control training	Exercises to work on the body's ability to maintain a correct alignment of the centre of gravity with respect to its body axis, controlling lateralization movements and promoting greater postural control and balance when seated. Exercises performed in different body positions with the aim of improving trunk control. For example: rotations, inclinations, Bobath exercises, etc.
Body-weight supported treadmill training	Gait training with a safety harness to prevent falls and increase the patient's security during the training. It is possible to use walkers or crutches if the patient requires it.
Standing training – Standing frame	Standing training aims to improve body schema, orthostatic adaptation training, lower extremity load tolerability training, etc.
Stairs, ramps and obstacles	Functional movement training upping stairs, ramps, and walking in a corridor with obstacles.

Gait training	Gait training with assistance or supervision by the physiotherapist with the aim of improve the pattern of gait. This activity also includes the integration training of orthosis, walkers or crutches use to prevent falls.
Transfer training	Activity that consists of changes of body position training in different situations. For example: supine to sitting, sitting in bed to sitting in a chair, sitting in bed to standing, etc. It requires a complex motor action of the body and a correct body schema.
Aerobic training	Cardiovascular training encompasses all those activities and exercises that increase heart rate and breathing by using large muscle groups in a repetitive and rhythmic way. Example: Cycle ergometer, treadmill, steps, circuits, etc.
Coordination and integration exercises of the affected side	Training to improve limb coordination and integration of the affected side of the body. For example: double task activities, dissociation of waists, bimanual tasks, etc.

OCCUPATIONAL ACTIVITIES

ACTIVITIES	DESCRIPTION OR EXAMPLES
Upper Limb movement patterns, coordination, and dexterity training	Actions or behaviours that the person uses to physically move and interact with tasks, objects and environments. Example: Pick up, move away, approach, reach, drop objects in different planes and directions. It includes planning, carrying out the sequences, as well as executing known and/or novel movements. Neuromusculoskeletal/Movement Related Functions: Joints (stability/mobility), muscle tone, muscle strength/endurance, motor reflexes, voluntary motor control and involuntary reactions.
Task-specific training	The repetitive practice of tasks related to objectives in certain functional situations and transferable to the person's natural environment. It seeks to improve and optimize learning and motor control, analysing and grading the purposeful activity based on the needs, abilities, and interests of the person. Example: Peel and cut food unimanually or place the clothespins with the affected upper limb at different heights and resistances.
Active-assisted tasks upper limb weight support	It is used when the affected upper limb does not have analytical mobility, we place it in a lower position using the suspension and it is the same patient, who, with the guidance of the occupational therapist, performs self-assisted exercises (helped with the functional upper limb), to promote mobility, provide proprioceptive information and favour the integration of the upper limb in the body scheme. Examples: Move a block with a two-hand grip (either on a horizontal surface or on an inclined plane) or grab and move cones at different heights and distances.
Postural control and orthosis use for affected upper limb	Activities to work on the body's ability to maintain a correct alignment of the centre of gravity with respect to its body axis, controlling lateralization movements and promoting greater postural control and balance when seated.<br>Also, we adapt different support products to the patient (arm supports, trays, tables to adapt to an armchair, sling for the affected arm only for use during ambulation). We make and adapt functional position splints to control spasticity and loss of alignment that some patients present in their affected hand.
Constraint Induced Movement Therapy (CIMT)	It consists of annulling/restricting/limiting the integration of the unaffected upper limb (with the use of gloves and slings or other elements) and promoting the movement of the affected upper limb, gradually and intensively through various exercises and activities.
Mirror therapy	It consists of the use of a mirror that creates a reflective illusion of the affected limb. The aim is to generate a "visual illusion"

	that makes one think that the hand seen in the mirror is not affected, "the brain is fooled", to achieve positive feedback in the motor cortex. It bases its operation on the "mirror neurons", located in the parietal cortex.
Virtual reality for upper limb movement training	Its operation is based on "mirror neurons", therapeutic activity within the use of new technologies, artificial intelligence computer programs, where the patient plays competitive and visually attractive games, which promotes the integration of the affected upper or lower limb, stimulating the brain of the affected areas.
Somatosensory rehabilitation	Enhance the neurological process that organizes the sensations and perception of the body, by stimulating the different tactile receptors. Example: Use of creams, brushes of different textures, vibration, discriminate objects by size, texture and temperature.
Cognitive and perceptual tasks	Intervention focused on improving higher functions (orientation, concentration, memory, attention, organization, and planning), which are involved in the performance of all basic and instrumental activities of daily living. About perceptual activities, it refers to the intervention of the occupational therapist in the visual disturbances that many stroke patients present (hemianopsia, diplopia, etc.). Examples: Use of calendars, schedules, "activate your mind" activities on the computer, cognitive games on the tablet, memory, design copy activities, psychomotor activities.
Activities of daily live training	Training in all basic activities of daily living (bed mobility, hygiene, dressing, grooming, feeding and transfers), aimed at achieving the best independent performance and being able to return home. Also, an initial intervention of the instrumental activities of daily life like cooking, doing housework, using money, using the mobile phone, etc.). Also, prior to hospital discharge, both the patient and the family are oriented on the basic support products that the patient requires at home, especially to promote greater independent performance of the patient, avoid overloading the caregiver and prevent a fall.
Stroke education	The Stroke Class is a space that aims to provide information, education and support for family members and patients who follow a rehabilitation program after suffered a stroke when they are discharged from hospital.

SPEECH THERAPY ACTIVITIES

ACTIVITIES	DESCRIPTION OR EXAMPLES
Verbal and written comprehension	Written comprehension, understand short texts and answer questions about the content. In the same way, oral comprehension is worked on by following verbal commands.
Oral production (articulation, volume, tone and prosody)	Oral production training by reading texts and repeating fragments.
Phonological processing	Training on phonetic awareness and discrimination using words. For example, locating the missing vowel or consonant in a word.
Morphosyntax's exercises	Grammatical structure: ordering the elements of a sentence, writing the verb tenses within the sentence, etc.
Functional communication	Functional communication training for the performance of their basic ADL and IADL. During the session, the patient must choose a topic and talk about it, explain what he has done the day before, etc.
Lexical evocation (free, phonetic phonological and semantic)	Evocation of words of the same semantic category, saying words that begin with a certain syllable or letter, etc.
Lingual praxis	Myofunctional training through tongue movements, tongue resistance and strength training, etc.
Cognition (memory, reasoning, logical reasoning and abstraction)	Order sequences of images, do crossword puzzles, find differences in different images, etc.
Respiratory Muscle Training (Orygen Dual Valve©)	Respiratory muscle training using Orygen-Dual Valve. Instructions for use: 5 cycles 3 times a day. (1 cycle: 5 inspirations + 5 expirations)
Sensitive and gustatory stimulation	Intraoral stimulation with vibration or taste discrimination.
Neuromuscular Electrical Stimulation (Vital Stym©)	VitalStim therapy is a specialized form of neuromuscular electrical stimulation (NMES) designed to treat swallowing disorders through muscle re-education. VitalStim therapy is delivered by a small current delivered to the motor nerves in the patient's throat through specially designed electrodes that cause the muscles responsible for swallowing to contract.
